# Transportation control and epidemic containment: empirical evidence from China's Wuhan lockdown

**DOI:** 10.3389/fpubh.2026.1777746

**Published:** 2026-05-26

**Authors:** Mengting Zhang, Yiwen Zhang, Feng Yu, Yanbin Wen

**Affiliations:** 1 China-CEEC Institute of Economic and Trade Cooperation, Ningbo University, Ningbo, China; 2 School of Business, Ningbo University, Ningbo, China; 3 School of Economics and Management, University of Science and Technology Beijing, Beijing, China; 4 School of Economics, Jiaxing University, Jiaxing, China

**Keywords:** COVID-19, epidemic containment, high-speed rail (HSR), human mobility, Wuhan lockdown

## Abstract

**Background:**

The rapid development of modern transportation networks, coupled with increasing urbanization and population mobility, presents a dual challenge for global public health systems: while enhancing connectivity and economic integration, these networks also accelerate the spatial dissemination of infectious diseases. The COVID-19 pandemic, which emerged in Wuhan, China, in late 2019, starkly illustrated this paradox. As a major national transportation hub with dense high-speed rail (HSR) connectivity, Wuhan exemplifies how modern transport infrastructure can facilitate the rapid intercity transmission of pathogens. The unprecedented lockdown of Wuhan on January 23, 2020, along with nationwide transportation controls, constituted a critical natural experiment for assessing the effectiveness of mobility restrictions in containing epidemic spread. This study provides timely empirical evidence on this pivotal public health intervention.

**Methods:**

Utilizing a multi-source dataset encompassing daily confirmed COVID-19 cases from 294 Chinese cities, HSR operational records, Baidu migration big data, and urban socioeconomic indicators, this study employs an econometric framework to evaluate the impact of transportation networks and controls on epidemic dynamics. Our empirical strategy involves cross-sectional and panel data analyses, with a focus on comparing cities with and without HSR connectivity before and after the Wuhan lockdown. We control for confounding factors such as city-level economic development, existing transportation infrastructure, and pre-lockdown population inflows from Wuhan. A series of robustness checks, including alternative model specifications, sample restrictions, placebo tests, and permutation simulations, are conducted to ensure the reliability of causal inferences.

**Results:**

Our analysis reveals that HSR connectivity served as a primary channel for the early intercity transmission of COVID-19. Cities connected to the HSR network, particularly those with direct links to Wuhan, experienced significantly higher numbers of confirmed cases. On January 30, 2020, HSR-connected cities (excluding Wuhan) had an average of 12.62 more cases than non-HSR cities, which accounted for an estimated excess of 2,852 cases nationally. The correlation between pre-lockdown population outflows from Wuhan and subsequent case distribution in destination cities was strongly positive. Importantly, the implementation of the Wuhan lockdown and associated transportation suspensions effectively disrupted this transmission pathway. The estimated effect of HSR on case counts diminished sharply immediately after the lockdown, underscoring the efficacy of mobility restrictions in curbing epidemic spread.

**Implications:**

This study demonstrates that large-scale transportation controls, exemplified by the Wuhan lockdown, can be a highly effective non-pharmaceutical intervention for containing emerging infectious diseases in their initial stages. The findings validate the scientific rationale behind China's containment strategy, which centered on source control and transmission route interruption. Beyond the immediate pandemic context, this research highlights the critical interplay between transportation planning and public health preparedness. It underscores the necessity for policymakers worldwide to integrate epidemic response mechanisms into the governance of highly mobile, interconnected urban systems. The evidence presented here offers a robust, data-driven rebuttal to politicized critiques of China's early response and provides valuable insights for designing coordinated, evidence-based mobility policies to mitigate future global health threats.

## 1 Introduction

A recent editorial in *The Lancet* noted that since the 1970s, outbreaks of zoonotic diseases—including Ebola, MERS, SARS, avian influenza, Zika, and COVID-19—have grown increasingly frequent. This trend stems from human encroachment into wildlife habitats, the international trade of exotic animals, and the expansion of global travel networks, all of which pose significant threats to public health and social stability. Concurrently, globalization, industrialization, and urbanization have fostered highly developed transportation systems and facilitated frequent population movements, making it a pressing global challenge to respond promptly and effectively to the spread of infectious diseases. Research has shown that the onset of pandemics can induce substantial economic anxiety, influenced by individuals' perceptions of disease risk (1). Historical evidence from the 1918 influenza pandemic further underscores the long-term socioeconomic disruptions that can arise from such health crises, highlighting the importance of timely and effective non-pharmaceutical interventions (2).

**Figure 1:**
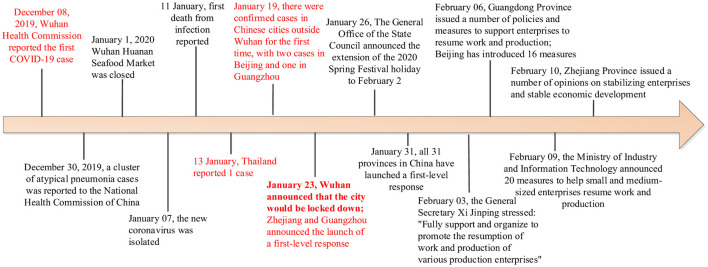
Timeline of key events in the initial COVID-19 outbreak in China.

On December 8, 2019, health officials reported unexplained cases of pneumonia in Wuhan, Hubei Province. By January 19, 2020, the National Health Commission of China had confirmed the first imported cases in Shenzhen and Beijing. The outbreak coincided with the Spring Festival travel rush, which aligns with the Lunar New Year and traditionally marks a peak period for interregional population movement in China. In an effort to curb the spread of the virus, the Wuhan municipal government announced a stringent city lockdown on January 23. This measure entailed the suspension of all bus, subway, ferry, and long-distance passenger transport operations within the city, as well as the temporary closure of airport and train station access to/from Wuhan. Simultaneously, other provinces across China promptly activated their highest-level public health emergency responses (as depicted in Figure 1). On March 11, 2020, based on an assessment of the geographic scope and severity of viral transmission, the World Health Organization declared COVID-19 a pandemic.

From an epidemiological and behavioral perspective, Wuhan, a metropolis of 11 million people and a major national transportation hub, was identified as the domestic epicenter of the outbreak. Its dense population and highly developed transport networks not only increased the risk of pathogen mutation but also complicated the tracing of transmission chains. In contemporary urban settings, the effectiveness of traditional containment measures, such as physical isolation and source control, faces new challenges. Rapid intercity mobility accelerates the spread of pathogens, while high population density facilitates viral mutation and cross-infection.

China took a series of timely prevention and control measures in the early stages of the outbreak. Notably, Wuhan entered lockdown on January 23, followed by strict nationwide restrictions on traffic and population movement. These actions proved effective in curbing the widespread transmission of COVID-19. Using a counterfactual approach, Shi and Qiu (3) estimated that China's containment efforts averted more than 1.4 million infections and 56,000 deaths. This rapid and decisive response prevented what could have been a large-scale public health crisis in a country of nearly 1.4 billion people during the high-mobility Spring Festival period. The Wuhan lockdown holds unprecedented significance in modern epidemic control and stands as the largest quarantine event in human history by population scale (4). This paper reviews China's early epidemic response, with a particular focus on the impact of the Wuhan lockdown on the spread of COVID-19. We examine the appropriateness and effectiveness of using transportation controls and regional isolation to limit intercity transmission. Our study relates to three main strands of literature:

First, research on measures to prevent and control epidemic spread. Historical experience demonstrates that physical isolation and containment of infection sources are among the most effective strategies during the early stages of an outbreak. Before the 1850s, epidemic prevention primarily relied on principles of “cleanliness” and “isolation and quarantine,” with an emphasis on identifying and isolating infected individuals. In the latter half of the 19th century, following the establishment of the germ theory of disease, the focus of public health policy shifted from isolating patients to interrupting the transmission of pathogens. Examples of isolation-based measures date back centuries: in 6th-century Europe, leprosy patients were confined to leprosaria; during the 14th-century Black Death, Milan avoided major outbreaks by quarantining affected households. In 1886, Japan contained a nationwide cholera epidemic by imposing area closures in Tokyo, which included forced quarantine and relocation of residents. Between 1910 and 1928, three plague outbreaks in northeastern China caused approximately 60,000 deaths. The spread of these outbreaks was exacerbated by the expansion of railroads and urbanization. In response, the government dispatched medical officers who implemented traffic controls, isolation of affected areas, sheltering of victims, and cremation of remains, measures that rapidly brought the epidemics under control. Modern epidemiology similarly identifies three core strategies for controlling infectious diseases: managing infection sources, interrupting transmission routes, and protecting susceptible populations (5). These principles have been applied in recent outbreaks; for instance, school closures were widely used during the 2009 influenza pandemic, while more targeted interventions such as restricting inter-district commuting, reducing individual activity, and enhancing PCR testing efficiency have been shown to effectively suppress urban COVID-19 outbreaks (6).

Second, literature concerning the impact of transportation infrastructure on virus transmission. While improved transport infrastructure facilitates the spatial mobility of people and goods, it also accelerates the spread of infectious diseases (7). Historical studies illustrate this dual role: Schmid et al. (8) note that the Silk Road trade in the 14th century enabled the introduction of the Black Death into Europe, while Yue et al. (9) identify major trade routes as key channels for plague dissemination in pre-industrial Europe. In a contemporary context, Djemai (10) examines road networks in five African countries and finds that enhanced road accessibility raises individuals' exposure to mobile populations, thereby increasing HIV transmission risk. Similarly, Zimram (11) uses 19th-century U.S. data to show that although transport development generally improves regional health outcomes, the resulting rise in population density can also facilitate epidemic spread. Much of the existing work on transport and health, however, remains correlational in nature [e.g., see (12–16)], often lacking robust causal identification and systematic evidence.

Third, literature focusing on the impact of population mobility on the spread of COVID-19. Utilizing nearly 3 million daily intercity movement records from Baidu Migration, Fang et al. (17) employed a difference-in-differences (DID) approach to evaluate the effect of the Wuhan lockdown on population flows. They found that the lockdown significantly reduced inbound, outbound, and intra-city mobility in Wuhan, lowering viral transmission by 76.64%, 56.35%, and 54.45%, respectively. Counterfactual simulations further confirmed that the lockdown effectively suppressed the spread of the virus. Similarly, Meng et al. (18) extracted vehicle outflow data from Wuhan to 245 cities using China's expressway toll collection system to examine the impact of Wuhan's expressway lockdown measures on the intercity transmission of COVID-19. Their results indicate that, at the national level, vehicle outflow from Wuhan was the primary factor influencing the spread of COVID-19 across Chinese cities. Extending the analysis from domestic to global scale, Kinasih et al. (19) demonstrate that large-scale population mobility during the Spring Festival travel season, together with international air travel, enabled COVID-19 to spread rapidly from Wuhan to other Chinese provinces and then across the world, eventually becoming a global public health emergency.

The lockdown of Wuhan constituted a critical intervention to contain the infection source at the onset of the COVID-19 outbreak. A central component of this measure was the suspension of outbound transportation from the city, including the halting of HSR services. Research in epidemiology and public health has demonstrated that the Wuhan lockdown, along with its associated restrictions on population mobility such as transport suspension, played a decisive role in controlling the spread of COVID-19 in China (4, 20). At the same time, a substantial body of literature has established that HSR exerts significant influence across multiple socioeconomic dimensions, including labor markets (21, 22), urban development (23–25), trade (26), and overall economic performance (23, 27, 28).

In summary, existing research presents at least three notable gaps. First, while numerous studies from both domestic and international sources have offered initial empirical evidence linking heterogeneous human mobility facilitated by various transportation modes to the spread of COVID-19, these investigations remain largely confined to the fields of epidemiology and public health. Second, within the domestic social sciences, recent literature on the prevention and control of COVID-19 and its broader impacts has been predominantly descriptive, with rigorous empirical analyses remaining scarce. Third, although HSR and other major transportation networks serve as primary channels for interregional and intercity population movement, research has focused largely on their effects on residents' health under normal socioeconomic conditions. There is a marked lack of studies examining the role of HSR in facilitating virus transmission and the effectiveness of transport-related interventions during public health emergencies.

This study empirically evaluates the effectiveness of China's epidemic containment strategies by focusing on the Wuhan lockdown and related transportation controls. Its marginal contributions are as follows.

First, we utilize real-time and accurate multi-source data, including COVID-19 case statistics from 294 Chinese cities, HSR operational data, city-level socioeconomic indicators, and Baidu migration records, to empirically analyze the effectiveness of China's epidemic response. This approach helps address the current scarcity of rigorous empirical research in the domestic social sciences and provides methodological insights for the assessment and management of future major infectious disease outbreaks.

Second, supported by mobile device location services and big data technologies, this paper provides new empirical evidence on the relationship between modern transportation networks and the spread of infectious diseases. Within the context of highly developed transport infrastructure and frequent urban mobility, we examine the impact of transportation restrictions on COVID-19 transmission. Our analysis reveals that HSR served as a key channel for the rapid interregional spread of the virus in the early stages of the pandemic. Moreover, control measures such as the Wuhan lockdown and the nationwide suspension of HSR services were effective in curbing intercity transmission. After controlling for other variables and accounting for the average incubation period of COVID-19 (7.5 days), we find that the 225 cities with access to HSR, excluding the initial epicenter Wuhan, reported significantly higher numbers of confirmed cases than the 69 cities without HSR. Specifically, the average differences in confirmed cases between these two groups on the first day (January 24), the second day (January 30), and the third day (February 6) after the lockdown were 0.777, 12.62, and 46.27, respectively, corresponding to cumulative differences of 175, 2,852, and 10,457 cases.

Third, from the perspective of the Wuhan lockdown and traffic controls, this study provides empirical evidence rooted in economics to support the scientific rationale behind China's containment strategies. The findings not only validate and extend epidemiological and public health research on the link between human mobility and the spread of COVID-19 but also respond to the politicization, stigmatization, and unfounded criticism directed at China's prevention and control efforts by certain countries.

## 2 HSR networks and epidemic spread in China

**Figure 2:**
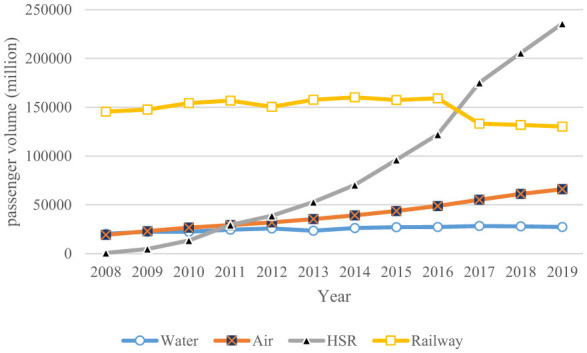
Changes in the share of passenger volume by transport mode, 2008–2019. Highway data are excluded from the current analysis. While highways account for the largest share of passenger transport volume in China, the available data aggregates all vehicular movement, including both intra-city and inter-city trips—such as those made by taxis and other local vehicles. Since this study focuses specifically on inter-city transportation, highway statistics are omitted to maintain analytical clarity and relevance. Source: Based on the data of transportation, post and telecommunications and software industry in the statistical yearbook of China.

The implementation of the Wuhan lockdown and the nationwide activation of the Level I public health emergency response relied centrally on strict traffic control measures within and between cities. Over the past two decades, China's transportation infrastructure has expanded rapidly, with HSR recognized as one of the country's “Four New Great Inventions” and attracting widespread attention. (1) The pace of HSR development has been remarkable. According to World Bank (43) data, between 2008 and 2019, China constructed over 35,000 kilometers of HSR lines, exceeding the total HSR mileage of all other countries combined. China now leads the world in HSR system technology, integration capability, operational mileage, operating speed, and the scale of ongoing construction. (2) The HSR network has become increasingly comprehensive. The system has evolved into the “Eight Vertical and Eight Horizontal”^1^ national network, connecting major economic zones such as the Yangtze River Delta, Pearl River Delta, and Bohai Rim through high-density rail corridors. This network integrates the eastern, central, western, and northeastern regions of the country. (3) The capacity and share of HSR in passenger transport continue to grow. Benefiting from affordable fares, high speeds, and extensive coverage, HSR holds a competitive advantage over road and air transport over distances up to 1,200 kilometers. The proportion of rail passenger traffic in China's total passenger transport increased from 5.1% in 2008 to 18.8% in 2018, with HSR's share within rail passenger traffic rising from 0.5% in 2008 to 64.4% in 2019 (see Figure 2). In 2019, China's railway system carried 3.57 billion passengers, of which 2.29 billion traveled by HSR, representing a year-on-year increase of 14.1%. These figures highlight the increasingly critical role of HSR in facilitating intercity population movement in China.

As a passenger-dedicated transport mode, HSR has played an increasingly vital role in facilitating regional and inter-city population mobility. In response to sustained travel demand, the “HSR Spring Festival travel rush” has emerged. During this period, HSR acts as the primary travel mode, greatly improving cross-regional transportation capacity. The Spring Festival travel rush, which coincides with the Lunar New Year, traditionally represents the annual peak in inter-regional population movement in China. This concentrated mobility underscores the critical importance of HSR in the context of epidemic prevention and control.^2^

**Figure 3:**
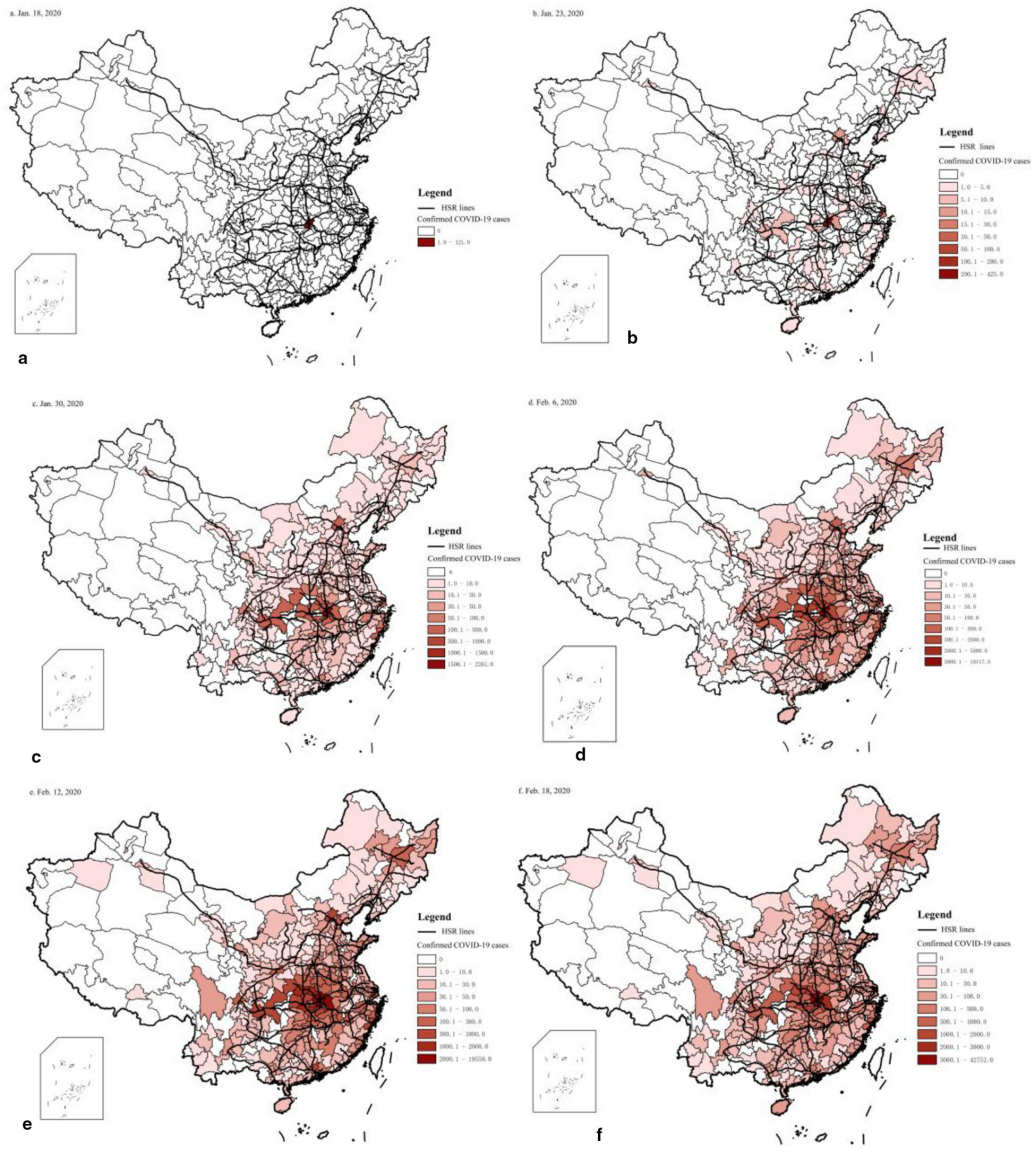
Spatial distribution of COVID-19 confirmed cases and HSR networks in China at six time points. The map is based on the standard map with the Map Approval Number GS(2016)1603 downloaded from the standard map service website (http://bzdt.ch.mnr.gov.cn/). **(a–f)** Correspond to January 18, January 23, January 30, February 6, February 12, and February 18, 2020, respectively. City colors (gradient shading) represent the number of confirmed COVID-19 cases, with darker shades indicating higher case counts. Lines depict high-speed rail (HSR) connections. The HSR lines shown are those already in operation as of 2019.

Wuhan, historically known as the “Thoroughfare to Nine Provinces,” is one of China's four major railway hubs. Its HSR network is particularly dense, anchored by the intersection of the Shanghai–Wuhan–Chengdu railway and the Beijing–Guangzhou HSR line. This strategic position has earned Wuhan the designation as the “heart of HSR” in China, underscoring the critical role HSR plays in facilitating both national and intercity passenger movement. Beyond rail, urban transit systems such as subways also represent high-risk environments for disease transmission, as demonstrated by modeling studies that incorporate passenger flow dynamics and asymptomatic carriers (29). Previous research has also highlighted the close relationship between transportation infrastructure and public health, particularly in highly mobile societies such as China (30). When epidemic data are mapped onto the HSR network, a distinct spatial correlation between rail connectivity and the spread of COVID-19 emerges (see Figure 3). This visual correspondence prompts several critical questions: did the HSR network actually shape the progression of the outbreak? If so, through which mechanisms did this influence operate? Furthermore, how can transportation planning and epidemic response be improved in the future? A systematic investigation into the role of HSR, a transportation mode of growing importance, in the dissemination of infectious diseases carries considerable theoretical weight and provides actionable insights for designing more effective epidemic prevention strategies.

## 3 Data and empirical strategy

### 3.1 Data sources

(1) COVID-19 data

**Figure 4:**
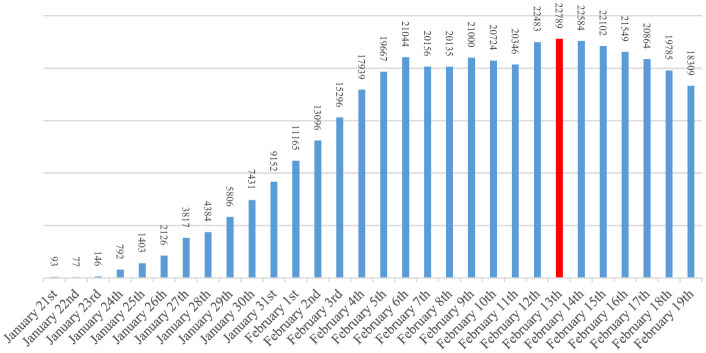
Number of people diagnosed with COVID-19 in China except Wuhan, January 21–February 19, 2020.

The COVID-19 data were collected from the official websites of the National Health Commission and provincial and municipal health commissions. We compiled daily records of confirmed cases, recoveries, and deaths across 294 prefecture-level cities in China from December 8, 2019, the date of the first reported case in Wuhan, through February 19, 2020. Figure 4 shows the number of confirmed COVID-19 cases in China (excluding Wuhan) during the period from January 21 to February 19, 2020.

To ensure consistency in reporting times, which varied across local health authorities, we used the daily release timestamp of the National Health Commission as the uniform data collection cutoff. A notable change in case classification criteria on February 12, 2020^3^, led to a sharp increase in reported cases. Therefore, our main analysis covers the period from December 8, 2019, to February 11, 2020. Data from February 12 to February 19 are retained for robustness checks.

The selection of this sample window is also motivated by two substantive considerations. First, the traffic control measures implemented during the Wuhan lockdown, which began on January 23, 2020, represent a decisive policy intervention. On that date, Wuhan suspended all intra-city and long-distance public transport, formally establishing traffic restrictions as a core epidemic containment tool. Second, following Wuhan's lockdown, numerous other cities activated Level I public health emergency responses, during which traffic control became widely adopted, allowing us to clearly observe the interplay between mobility restrictions and epidemic dynamics.

(2) China HSR data

HSR data in this study were obtained from textual information published in news reports and official announcements, including sources such as the China Railway Yearbook, the website of China Railway Corporation (currently China State Railway Group), and the National Railway Administration of the People's Republic of China. Details regarding the planning, construction, and opening dates of each HSR line were also collected. As of the end of 2019, HSR services were available in 174 prefecture-level cities in China, covering more than 58.6 percent of all such cities in the country. After merging with other economic datasets, the final sample used in this study includes 294 cities, including both prefecture-level and county-level cities. Among them, 226 cities, including Wuhan, are connected to the HSR network, while 68 cities are not. Additionally, based on timetable information from the official ticketing website 12,306, we identified 118 cities that could be reached directly from Wuhan via HSR. Daily frequencies of HSR departures from each of these cities were also compiled.

(3) Urban development data

City-level economic and social development indicators were collected from recent editions of the *City Statistical Yearbook*. These include data on regional foreign trade, gross regional product (GDP), population size, government fiscal revenue, total industrial output, as well as measures of infrastructure and transportation development.

(4) Baidu migration data

Baidu Migration Data are derived from Baidu Maps and third-party user location statistics. The platform provides a real-time, dynamic visualization of daily population flows between regions, accurately recording the movement trajectories of hundreds of millions of people. In 2020, the Baidu Maps Migration Big Data Platform employed three main indicators to capture mobility intensity from different perspectives^4^ the City Daily Migration Index, the City Daily Migration Index, and the City Daily Internal Travel Intensity. The calculation of migration indices is based on the identification of inter-city travel. A migration event is recorded when an individual leaves their city of residence or remains in a non-resident city for more than 1 day. This is then classified as an outbound movement from the 'departure city. Correspondingly, if an individual stays in a destination city for over 4 h, this is defined as an inbound movement to the “arrival city.” ^5^

Table 1 presents descriptive statistics for the main variables used in this study, based on cross-sectional data as of January 30, 2020. The sample is divided into cities with and without HSR connections. The definitions and measurements of the main variables are as follows:

(1) NCP_rate refers to the number of confirmed COVID-19 cases divided by the number of days since the outbreak began. For the 225 HSR cities (excluding Wuhan), the average daily confirmed case rate as of January 30, 2020, was 0.971, with a maximum of 68.52 and a minimum of 0.
(2) NCP_case denotes the total number of confirmed cases. Among the 225 HSR cities, the mean number of confirmed cases was 32.14, ranging from 0 to 2,261. For cities without HSR, the average was 2.449 cases, with a maximum of 17 and a minimum of 0.
(3) HSR is a binary indicator for whether a city had operational HSR services as of 2019. This serves as the core explanatory variable in the analysis.
(4) HSR_zd indicates whether a city had direct HSR connectivity to Wuhan.
(5) HSR intensity measures the daily frequency of HSR departures from each city, based on 2019 schedules. The average daily departure frequency across HSR cities was 186.3, with a maximum of 1,226.
(6) Airport is a dummy variable equal to 1 if a city had a civil aviation airport.
(7) Highway intensity represents road network density, calculated as the total graded highway mileage divided by the city's area and expressed in logarithmic form.
(8) Railway is a binary variable indicating whether a city was served by conventional rail.
(9) Lngdp_p is the natural logarithm of city-level GDP per capita.
(10) Lncity_p is the natural logarithm of the inflow population from Wuhan during the week prior to the city's lockdown (January 17–23, 2020).

**Table 1:** Descriptive statistics of main variables (cross-sectional data as of January 30).

Variable	Variable description	*N*	mean	sd	max	min
Panel A: opening of HSR cities						
NCP_rate	Number of confirmed cases/day	226	0.971	4.784	68.52	0
NCP_case	Number of confirmed cases	226	32.04	157.9	2261	0
Cure_case	Number of people cured	226	0.407	3.645	54	0
Death_case	Number of deaths	226	0.721	8.625	129	0
HSR	Whether the HSR has been opened	226	1	0	1	1
HSR_zd	Non-stop HSR to Wuhan	226	0.527	0.500	1	0
HSR intensity	The frequency of HSR	226	186.3	202.1	1226	0
Airport	Availability of airports	226	0.531	0.500	1	0
Highway intensity	Highway density	226	0.0325	0.492	0.811	−2.370
Railway	Availability of railroads	226	1	0	1	1
Lngdp_p	GDP per capita taken as logarithm	226	10.81	0.503	12.28	9.384
Lncity_p	The population left Wuhan one week before Wuhan lockdown taken as a logarithm	226	0.539	0.739	4.287	0
Panel B: cities without HSR						
NCP_rate	Number of confirmed cases/day	68	0.0713	0.103	0.515	0
NCP_case	Number of confirmed cases	68	2.353	3.407	17	0
Cure_case	Number of people cured	68	0	0	0	0
Death_case	Number of deaths	68	0.0147	0.121	1	0
HSR	Whether the HSR has been opened	68	0	0	0	0
HSR_zd	Non-stop HSR to Wuhan	68	0	0	0	0
HSR intensity	The frequency of HSR	68	0	0	0	0
Airport	Availability of airports	68	0.618	0.490	1	0
Highway intensity	Highway density	68	−0.660	0.863	0.691	−3.197
Railway	Availability of railroads	68	0.868	0.341	1	0
Lngdp_p	GDP per capita taken as logarithm	68	10.49	0.573	12.01	9.237
Lncity_p	The population left Wuhan one week before Wuhan lockdown taken as a logarithm	68	0.112	0.149	0.718	0

### 3.2 Empirical specification

The transmission of infectious diseases occurs when susceptible individuals come into contact with infected persons. The operation of HSR can theoretically elevate the probability of such contacts, thereby raising the potential for secondary infections. Recent evidence from China suggests that early diagnostic confirmation of cases can significantly reduce both prevalence and mortality, underscoring the importance of swift public health responses in mitigating outbreak severity (31). This study empirically investigates the impact of HSR connectivity on COVID-19 transmission, adapting the modeling approach employed by Lin (32). The baseline regression specification is as follows:


$$\begin{array}{c} Case_{c,t}=\lambda_{1}HSR_{c,2019}+\lambda_{2}Z_{c}+u_{c} \end{array}$$ (1)

where, *Case*_*c, t*_ denotes the number of confirmed COVID-19 cases in city *c* at time *t*. The main explanatory variable, denoted as *HSR*_*c*, 2019_, is a binary indicator that equals 1 if city *c* had operational HSR services in 2019, and 0 otherwise. The coefficient λ_1_captures the estimated effect of HSR connectivity on COVID-19 transmission. A positive and statistically significant λ_1_ suggests that HSR operation is associated with an increase in infections, while a negative and significant coefficient would indicate a mitigating effect.

The vector *Z*_*c*_ represents a set of city-level control variables, which include GDP per capita, the presence of an airport, and highway density. These controls are included to account for potential confounding factors at the city level. Due to data availability, GDP per capita and highway density are measured as of 2016. Additionally, indicator variables for whether a city had an airport or a conventional railway in 2019 are incorporated within *Z*_*c*_. The coefficient vector λ_2_ captures the marginal effects of the city-level control variables *Z*_*c*_ on confirmed COVID-19 cases, conditional on HSR connectivity and other included factors. Given that the primary variables in this study are measured at the city level, we follow the approach of Bertrand et al. (33) and cluster standard errors at the city level. This adjustment addresses potential issues of heteroskedasticity and spatial correlation in the error terms.

The empirical strategy of this study is structured as follows. (1) Wuhan is excluded from the regression analysis since COVID-19 spread outward from Wuhan as established in prior literature (17). Moreover, Wuhan implemented a comprehensive lockdown on January 23, 2020. Considering that the average incubation period of COVID-19 is approximately 7.5 days (34), the baseline regression employs data from January 30, 2020, to align with the expected timing of symptom onset following the initial outbreak and lockdown. (2) Second, to address potential endogeneity, we incorporate a set of city-level control variables to mitigate bias arising from omitted variables. Given that the morbidity and transmissibility of COVID-19 are largely exogenous to local economic or transport conditions, reverse causality is not a primary concern in this context. (3) We conduct a series of robustness checks and placebo tests to validate the consistency and reliability of the findings. (4) To further examine the policy effect of the Wuhan lockdown, we also analyze the influence of HSR connectivity on the volume of outbound travelers from Wuhan during the Spring Festival travel period.

## 4 Empirical results and their analysis

### 4.1 Baseline regression

Table 2 presents the regression results examining the relationship between HSR and COVID-19 infection. Control variables, including other transportation infrastructure indicators and city-level GDP per capita, are introduced stepwise across the models. Model (3) reports the baseline specification. The coefficient on the HSR indicator is positive and statistically significant, with a value of 12.62. This implies that, as of January 30, 2020, and holding other factors constant, cities with HSR service had on average 12.62 more confirmed COVID-19 cases than cities without HSR. Aggregating across all HSR cities, this corresponds to an excess of 2,852 confirmed cases, representing 37.7% of the national total (7,568 cases) recorded on that date. In summary, the baseline regression results provide clear evidence that HSR passenger transport served as a major channel for the rapid regional and inter-city spread of COVID-19 during the early phase of the pandemic.

**Table 2:** Baseline regression results for the effect of HSR on COVID-19 infection on January 30.

Variable	(1)	(2)	(3)
	NCP_case	NCP_case	NCP_case
HSR	19.78^***^ (5.61)	13.10^***^ (5.03)	12.62^***^ (4.03)
Airport		−0.0483 (−0.01)	−0.551 (−0.11)
Highway intensity		11.55^***^ (4.35)	11.22^***^ (3.79)
Railway		−9.772^*^ (−1.77)	−10.47^**^ (−2.00)
Lngdp_p			2.431 (0.52)
Cons	2.353^***^ (5.72)	18.48^**^ (2.28)	−6.327 (−0.12)
*N*	293	293	293
*R*-squared	0.0320	0.0524	0.0531

*T*-values are inside parentheses. ^*^p < 0.1, ^**^p < 0.05, ^***^p < 0.01.

### 4.2 Robustness checks

To ensure the reliability of our baseline findings, we conduct a series of robustness tests.

1. Alternative dependent variables

In the baseline regression, the total number of confirmed cases is used as the dependent variable. For robustness, we also employ two alternative measures: the daily average confirmed cases (total cases divided by the number of days since the outbreak, NCP_rate) and the confirmed cases per capita (total cases divided by city population, NCP_per). The results, presented in columns (1) and (2) of Table 3, show that the positive and significant effect of HSR remains consistent across both alternative specifications.

2. Direct HSR connection to Wuhan

**Table 3:** Robustness test of HSR's impact on COVID-19 infections (different indicators).

Variable	(1)	(2)	(3)	(4)
	NCP_rate	NCP_per	NCP_case	NCP_case
HSR	0.382^***^ (4.03)	0.0266^***^ (3.33)		
HSR_zd			17.61^**^ (2.52)	
HSR intensity				0.0548^***^ (4.32)
Airport	−0.0167 (−0.11)	−0.0251^*^ (−1.79)	−0.472 (−0.10)	−3.196 (−0.64)
Highway intensity	0.340^***^ (3.79)	0.0187^**^ (2.32)	10.10^***^ (4.36)	9.315^***^ (3.02)
Railway	−0.317^**^ (−2.00)	−0.0276^**^ (−2.05)	−5.024 (−1.17)	−3.351 (−0.87)
Lngdp_p	0.0737 (0.52)	0.00809 (0.78)	0.285 (0.05)	−3.598 (−0.74)
Cons	−0.192 (−0.12)	−0.0233 (−0.22)	13.84 (0.23)	54.69 (0.98)
*N*	293	293	293	293
*R*-squared	0.0531	0.0444	0.0717	0.0768

T-values are inside parentheses. ^*^p < 0.1, ^**^p < 0.05, ^***^p < 0.01.

Given that COVID-19 originated in Wuhan, cities with direct HSR links to Wuhan might have been more exposed to early infections. We therefore differentiate between cities with and without direct HSR connections to Wuhan. As shown in column (3) of Table 3, the coefficient on the direct-connection indicator is 17.61 and statistically significant, indicating that cities directly linked to Wuhan by HSR experienced substantially more cases. Additionally, column (4) reports that the frequency of HSR services (HSR intensity)^6^ also shows a positive and significant coefficient (0.0548), implying that each additional daily HSR departure is associated with an increase of approximately 0.055 confirmed cases.

3. Excluding Hubei province

Since Hubei Province accounted for 58.22% of national cases as of January 30, we re-estimate the model after removing all cities in Hubei. The results, reported in Table 4, continue to show a statistically significant positive effect of HSR, confirming that the main findings are not driven solely by the epidemic's epicenter.

4. Adjusting for epidemiological contact history

**Table 4:** Robustness test for the effect of HSR on COVID-19 infection (Hubei Province sample deleted).

Variable	(1)	(2)	(3)	(4)	(5)
	NCP_case	NCP_rate	NCP_per	NCP_case	NCP_case
HSR	5.454^***^ (4.03)	0.165^***^ (4.03)	0.00774^***^ (2.81)		
HSR_zd				9.524^***^ (4.05)	
HSR intensity					0.0527^***^ (5.40)
Airport	7.702^***^ (3.56)	0.233^***^ (3.56)	0.00444^*^ (1.70)	7.595^***^ (3.59)	4.998^***^ (2.66)
Highway intensity	6.639^***^ (4.57)	0.201^***^ (4.57)	0.00653^***^ (3.98)	5.809^***^ (4.76)	3.264^***^ (2.73)
Railway	−5.271 (−1.53)	−0.160 (−1.53)	−0.00780^*^ (−1.93)	−3.027 (−1.04)	−1.271 (−0.94)
Lngdp_p	7.098^***^ (3.25)	0.215^***^ (3.25)	0.00989^***^ (4.11)	5.751^***^ (2.92)	0.467 (0.39)
Cons	−67.94^***^ (−3.00)	−2.059^***^ (−3.00)	−0.0857^***^ (−3.40)	−55.32^***^ (−2.69)	−2.879 (−0.23)
*N*	282	282	282	282	282
*R*-squared	0.167	0.167	0.147	0.202	0.330

T-values are inside parentheses. ^*^p < 0.1, ^**^p < 0.05, ^***^p < 0.01.

Based on Guan et al. (35), about 74% of confirmed cases in early 2020 had a history of contact with Wuhan. To account for this, we adjust the confirmed case counts by multiplying them by 74% as a proxy for Wuhan-related transmissions. The regression results using this adjusted measure, presented in Table 5, remain robust and consistent with the baseline estimates.

5. Excluding major central cities

**Table 5:** Robustness test of HSR impact on COVID-19 infection (based on epidemiological indicators).

Variable	(1)	(2)	(3)	(4)	(5)
	NCP_case	NCP_rate	NCP_per	NCP_case	NCP_case
HSR	9.332^***^ (4.02)	0.283^***^ (4.02)	0.0197^***^ (3.31)		
HSR_zd				13.03^**^ (2.52)	
HSR intensity					0.0405^***^ (4.32)
Airport	−0.505 (−0.14)	−0.0153 (−0.14)	−0.0188^*^ (−1.81)	−0.349 (−0.10)	−2.365 (−0.64)
Highway intensity	8.249^***^ (3.77)	0.250^***^ (3.77)	0.0137^**^ (2.31)	7.471^***^ (4.36)	6.893^***^ (3.02)
Railway	−7.714^**^ (−2.00)	−0.234^**^ (−2.00)	−0.0204^**^ (−2.03)	−3.718 (−1.17)	−2.480 (−0.87)
Lngdp_p	1.878 (0.54)	0.0569 (0.54)	0.00614 (0.81)	0.211 (0.05)	−2.662 (−0.74)
Cons	−5.482 (−0.14)	−0.166 (−0.14)	−0.0189 (−0.25)	10.24 (0.23)	40.47 (0.98)
*N*	293	293	293	293	293
*R*-squared	0.0532	0.0532	0.0445	0.0717	0.0768

T-values are inside parentheses. ^*^p < 0.1, ^**^p < 0.05, ^***^p < 0.01.

Prior literature notes that regions with stronger economic bases tend to have better transport infrastructure, and HSR planning often prioritizes connecting major administrative centers. To mitigate potential bias from the higher economic activity and inherent mobility of such cities, we exclude municipalities directly under the central government, provincial capitals, and sub-provincial cities from the sample. The results, shown in Table 6, continue to support a positive and significant role of HSR in COVID-19 transmission.

6. Using panel data from different dates

**Table 6:** Robustness test for the effect of HSR on COVID-19 infection (large cities deleted).

Variable	(1)	(2)	(3)	(4)	(5)
	NCP_case	NCP_rate	NCP_per	NCP_case	NCP_case
HSR	12.39^***^ (4.00)	0.376^***^ (4.00)	0.0281^***^ (3.43)		
HSR_zd				15.78^**^ (2.08)	
HSR intensity					0.0514^**^ (2.30)
Airport	−3.816 (−0.75)	−0.116 (−0.75)	−0.0218 (−1.59)	−2.739 (−0.57)	−3.700 (−0.73)
Highway intensity	9.736^***^ (3.18)	0.295^***^ (3.18)	0.0201^**^ (2.23)	9.432^***^ (3.85)	9.560^***^ (3.01)
Railway	−8.604^*^ (−1.91)	−0.261^*^ (−1.91)	−0.0292^**^ (−1.99)	−3.584 (−0.91)	−3.360 (−0.83)
Lngdp_p	−2.680 (−0.53)	−0.0812 (−0.53)	0.0105 (0.84)	−3.354 (−0.61)	−4.499 (−0.84)
Cons	46.50 (0.80)	1.409 (0.80)	−0.0488 (−0.38)	51.97 (0.86)	64.69 (1.06)
*N*	259	259	259	259	259
*R*-squared	0.0450	0.0450	0.0446	0.0569	0.0463

T-values are inside parentheses. ^*^p < 0.1, ^**^p < 0.05, ^***^p < 0.01.

Considering the average incubation period of COVID-19 (7.5 days), we examine outcomes on alternative dates: January 24 (one day after the lockdown), February 6 (14 days after lockdown), February 12, and February 18. Table 7 shows that on January 24, the HSR coefficient is 0.777, about 6.2% of the baseline estimate from January 30. This suggests that most infections detectable soon after the lockdown originated from outflows from Wuhan before January 17. By February 6 (Table 8), the coefficient rises to 46.27, reflecting the accumulation of infections over time, including secondary and tertiary transmissions. Results for February 12 and February 18 (Tables 9, 10) further confirm that the positive association between HSR and case counts remains qualitatively unchanged even after the adjustment of diagnostic criteria in mid-February.

7. Controlling for day fixed effects

**Table 7:** Robustness test for the effect of HSR on COVID-19 infection (January 24^*th*^).

Variable	(1)	(2)	(3)	(4)	(5)
	NCP_case	NCP_rate	NCP_per	NCP_case	NCP_case
HSR	0.777^***^ (3.61)	0.0288^***^ (3.61)	0.0288^***^ (3.61)		
HSR_zd				1.572^***^ (3.94)	
HSR intensity					0.00977^***^ (3.17)
Airport	1.141^***^ (3.04)	0.0423^***^ (3.04)	0.0423^***^ (3.04)	1.146^***^ (3.08)	0.661^**^ (2.23)
Highway intensity	0.782^**^ (2.54)	0.0290^**^ (2.54)	0.0290^**^ (2.54)	0.605^**^ (2.44)	0.117 (0.55)
Railway	−0.970^*^ (−1.70)	−0.0359^*^ (−1.70)	−0.0359^*^ (−1.70)	−0.635 (−1.29)	−0.336 (−1.27)
Lngdp_p	1.393^***^ (2.94)	0.0516^***^ (2.94)	0.0516^***^ (2.94)	1.164^***^ (2.64)	0.161 (0.54)
Cons	−13.85^***^ (−2.82)	−0.513^***^ (−2.82)	−0.513^***^ (−2.82)	−11.78^**^ (−2.56)	−1.832 (−0.58)
N	293	293	293	293	293
R-squared	0.118	0.118	0.118	0.150	0.290

T-values are inside parentheses. ^*^p < 0.1, ^**^p < 0.05, ^***^p < 0.01.

**Table 8:** Robustness test for the effect of HSR on COVID-19 infection (February 6^*th*^).

Variable	(1)	(2)	(3)	(4)	(5)
	NCP_case	NCP_rate	NCP_per	NCP_case	NCP_case
HSR	46.27^***^ (3.63)	1.157^***^ (3.63)	0.103^***^ (2.92)		
HSR_zd				64.00^**^ (2.27)	
HSR intensity					0.155^***^ (3.47)
Airport	−16.51 (−0.85)	−0.413 (−0.85)	−0.119^**^ (−2.09)	−16.22 (−0.84)	−23.96 (−1.18)
Highway intensity	35.78^***^ (3.07)	0.894^***^ (3.07)	0.0596^*^ (1.86)	31.78^***^ (3.47)	32.68^***^ (2.68)
Railway	−34.93^*^ (−1.94)	−0.873^*^ (−1.94)	−0.0857^*^ (−1.75)	−14.96 (−1.03)	−10.21 (−0.71)
Lngdp_p	−0.305 (−0.02)	−0.00764 (−0.02)	0.0159 (0.40)	−8.065 (−0.38)	−16.30 (−0.81)
Cons	73.66 (0.35)	1.841 (0.35)	0.0482 (0.12)	146.6 (0.62)	238.8 (1.05)
*N*	293	293	293	293	293
*R*-squared	0.0398	0.0398	0.0404	0.0552	0.0485

T-values are inside parentheses. ^*^p < 0.1, ^**^p < 0.05, ^***^p < 0.01.

**Table 9:** Robustness test for the effect of HSR on COVID-19 infection (February 12^*th*^).

Variable	(1)	(2)	(3)	(4)	(5)
	NCP_case	NCP_rate	NCP_per	NCP_case	NCP_case
HSR	63.01^***^ (3.55)	1.370^***^ (3.55)	0.140^***^ (2.80)		
HSR_zd				89.70^**^ (2.28)	
HSR intensity					0.196^***^ (3.20)
Airport	−27.99 (−1.02)	−0.609 (−1.02)	−0.189^**^ (−2.10)	−27.60 (−1.01)	−37.33 (−1.30)
Highway intensity	50.54^***^ (3.07)	1.099^***^ (3.07)	0.0968^*^ (1.80)	44.53^***^ (3.43)	47.70^***^ (2.74)
Railway	−49.38^*^ (−1.90)	−1.073^*^ (−1.90)	−0.138 (−1.52)	−22.18 (−1.05)	−16.21 (−0.74)
Lngdp_p	−1.217 (−0.05)	−0.0264 (−0.05)	0.0338 (0.52)	−12.29 (−0.42)	−20.83 (−0.74)
Cons	115.6 (0.40)	2.514 (0.40)	−0.0130 (−0.02)	219.3 (0.68)	319.7 (1.00)
*N*	293	293	293	293	293
*R*–squared	0.0405	0.0405	0.0390	0.0564	0.0464

T-values are inside parentheses. ^*^p < 0.1, ^**^p < 0.05, ^***^p < 0.01.

**Table 10:** Robustness test for the effect of HSR on COVID-19 infection (February 18^*th*^).

Variable	(1)	(2)	(3)	(4)	(5)
	NCP_case	NCP_rate	NCP_per	NCP_case	NCP_case
HSR	73.80^***^ (3.49)	1.419^***^ (3.49)	0.163^***^ (2.72)		
HSR_zd				108.5^**^ (2.31)	
HSR intensity					0.213^***^ (2.91)
Airport	−40.55 (−1.24)	−0.780 (−1.24)	−0.254^**^ (−2.08)	−40.08 (−1.23)	−50.71 (−1.48)
Highway intensity	60.09^***^ (3.02)	1.156^***^ (3.02)	0.130^*^ (1.72)	52.29^***^ (3.41)	58.12^***^ (2.75)
Railway	−61.55^*^ (−1.93)	−1.184^*^ (−1.93)	−0.193 (−1.43)	−29.70 (−1.15)	−23.18 (−0.83)
Lngdp_p	−3.524 (−0.12)	−0.0678 (−0.12)	0.0516 (0.56)	−17.17 (−0.49)	−24.37 (−0.71)
Cons	165.6 (0.49)	3.185 (0.49)	−0.0855 (−0.10)	292.9 (0.76)	384.3 (1.00)
*N*	293	293	293	293	293
*R*-squared	0.0412	0.0412	0.0369	0.0579	0.0450

T-values are inside parentheses. ^*^p < 0.1, ^**^p < 0.05, ^***^p < 0.01.

Finally, we re-estimate the model using the full daily panel from December 8, 2019, to February 18, 2020, while including day fixed effects. The results, presented in Table 11, continue to show a statistically significant positive coefficient on HSR, indicating that the estimated relationship is robust to the control of daily common shocks.

**Table 11:** Robustness check for the effect of HSR on COVID-19 infection (December 8^*th*^ to February 18^*th*^).

Variable	(1)	(2)	(3)	(4)	(5)
	NCP_case	NCP_rate	NCP_per	NCP_case	NCP_case
HSR	19.69^***^ (3.64)	0.451^***^ (3.67)	0.0435^***^ (2.89)		
HSR_zd				27.99^**^ (2.34)	
HSR intensity					0.0640^***^ (3.37)
Airport	−8.126 (−0.98)	−0.176 (−0.93)	−0.0577^**^ (−2.11)	−8.002 (−0.97)	−11.19 (−1.29)
Highway intensity	15.87^***^ (3.17)	0.364^***^ (3.20)	0.0301^*^ (1.85)	13.99^***^ (3.57)	14.73^***^ (2.78)
Railway	−15.78^**^ (−1.97)	−0.362^**^ (−1.98)	−0.0441 (−1.60)	−7.281 (−1.12)	−5.325 (−0.80)
Lngdp_p	0.153 (0.02)	0.00978 (0.06)	0.0112 (0.56)	−3.300 (−0.37)	−6.371 (−0.74)
Cons	5.066 (0.06)	0.0439 (0.02)	−0.0756 (−0.39)	37.42 (0.39)	72.62 (0.77)
Day Fixed Effect	YES	YES	YES	YES	YES
*N*	15236	15236	15236	15236	15236
*R*-squared	0.0716	0.0711	0.0569	0.0768	0.0740

T-values are inside parentheses. ^*^p < 0.1, ^**^p < 0.05, ^***^p < 0.01.

Overall, the results across these diverse robustness checks consistently support the conclusion that HSR connectivity played a significant role in facilitating the spatial spread of COVID-19 in the early stages of the pandemic.

### 4.3 Placebo test

We conduct a placebo test by substituting the dependent variable from confirmed COVID-19 cases to the numbers of recoveries and deaths. Djemai (10) suggests that improved transportation infrastructure may reduce the spread of certain diseases, such as HIV, by enhancing access to preventive resources and health information. However, COVID-19 is a highly contagious respiratory infection whose transmission is more directly tied to human mobility rather than to healthcare access *per se*, particularly in the acute phase when isolation and quarantine are the primary control measures. Consequently, transportation infrastructure is less likely to systematically influence COVID-19 outcomes such as recovery or mortality in the short term.

Therefore, we hypothesize that the number of recoveries and deaths should be largely determined by individual immune responses and local healthcare capacity, rather than by whether a city is connected to the HSR network. The regression results reported in Table 12 confirm this expectation: the coefficients on the HSR indicator are statistically insignificant for both recovery counts and death counts. This finding reinforces the validity of our main results, as it suggests that the observed association between HSR and confirmed cases is not merely an artifact of general infrastructure effects, but rather reflects the specific role of HSR in facilitating population mobility and virus transmission.

**Table 12:** Empirical results on the effect of HSR on cure and death of COVID-19.

Variable	(1)	(2)
	Cure_case	Death_case
HSR	0.0856 (0.91)	0.0938 (0.69)
Airport	0.0517 (0.71)	−0.151 (−1.44)
Highway intensity	0.0663 (1.07)	0.101 (1.14)
Railway	−0.108 (−0.49)	−0.0919 (−0.29)
Lngdp_p	0.183^***^ (2.65)	−0.0917 (−0.92)
Cons	−1.812^**^ (−2.46)	1.216 (1.14)
N	293	293
R-squared	0.0481	0.0231

T-values are inside parentheses. ^*^p < 0.1, ^**^p < 0.05, ^***^p < 0.01.

**Figure 5:**
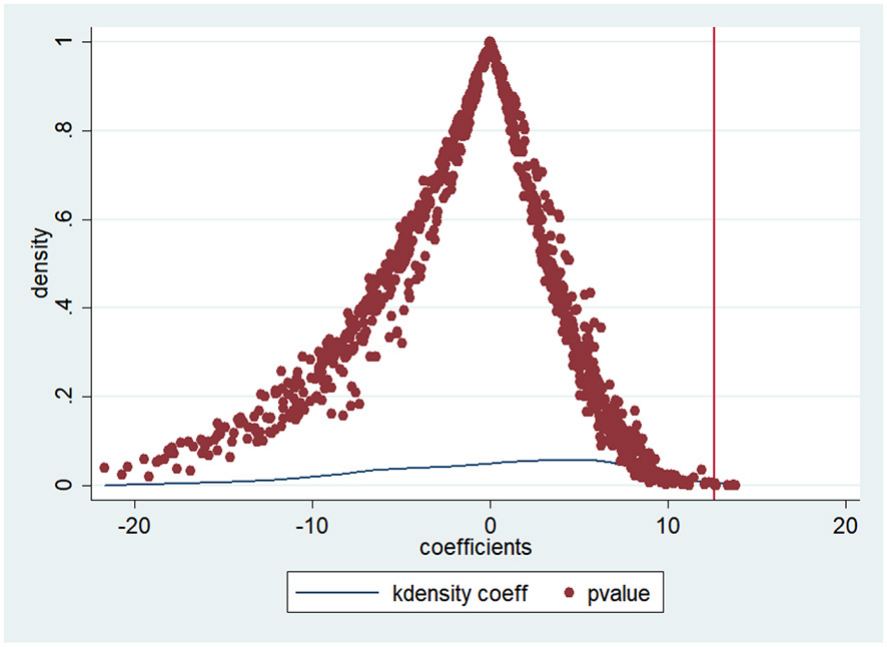
Simulation results of randomly assigned HSR cities.

Second, to address potential omitted variable bias, we perform a permutation test by randomly assigning the “treatment” status of HSR connectivity across cities. Following methods used in Li et al. (36) and Shen et al. (37), we randomly select each year the same number of cities as those that actually opened HSR to form a placebo treatment group. This process is repeated 1,000 times, each time re-estimating Equation (1) with the simulated treatment indicator, and the specific results are shown in Figure 5. The distribution of the resulting placebo coefficients is centered around zero, with a mean of −0.154. In contrast, our baseline estimate of 12.62 lies far in the right tail of this distribution. Only 8 out of the 1,000 simulated coefficients exceed 12.62 and are statistically significant at the 10% level, implying that the probability of observing a coefficient as large as ours by chance is merely 0.8%. In other words, our estimated effect of HSR on COVID-19 transmission is highly unlikely to be driven by unobserved city characteristics. Figure 5 visually summarizes the distribution of placebo coefficients and the position of our baseline estimate, reinforcing the robustness of the causal interpretation.

## 5 Further discussion: the scientific basis for the Wuhan lockdown

Epidemic spread is governed by three key factors: the infectious source, the transmission route, and the susceptible population. According to World Health Organization estimates, the basic reproduction number (R0)^7^ of the novel coronavirus ranges between 1.5 and 3.5, which is higher than that of SARS and accompanied by a longer incubation period. Wu et al. (38) estimated R0 to be 2.68. To contain transmission driven by population mobility, Wuhan implemented a citywide lockdown on January 23, suspending all transport links with other cities and formally establishing traffic control as a core public health intervention. Many other cities soon adopted similar transport restrictions, leading to the gradual nationwide suspension of high-speed rail (HSR) services. Previous studies, along with the findings presented in earlier sections of this paper, have established a correlation between outflows from Wuhan before the lockdown and the number of confirmed cases in destination cities^8^. Below, we further examine the specific role of the Wuhan lockdown in early epidemic containment.

First, using cumulative case data from provincial and municipal health commission websites (as of February 11, 2020) and Baidu Maps Spring Festival Migration Big Data (covering December 8, 2019 to January 23, 2020), we conduct a correlation analysis between population movement from Wuhan and COVID-19 spread. The procedure consists of three steps:

(1) Based on Baidu migration data, we calculate the average daily proportion of outbound travelers from Wuhan to each destination city during the week before the lockdown (January 17–23, 2020).
(2) We multiply these proportions by the estimated total outflow of 5 million people from Wuhan during that period to approximate the number of arrivals from Wuhan in each destination city.
(3) We then fit these estimated inflows against the cumulative confirmed cases in each city.

**Figure 6:**
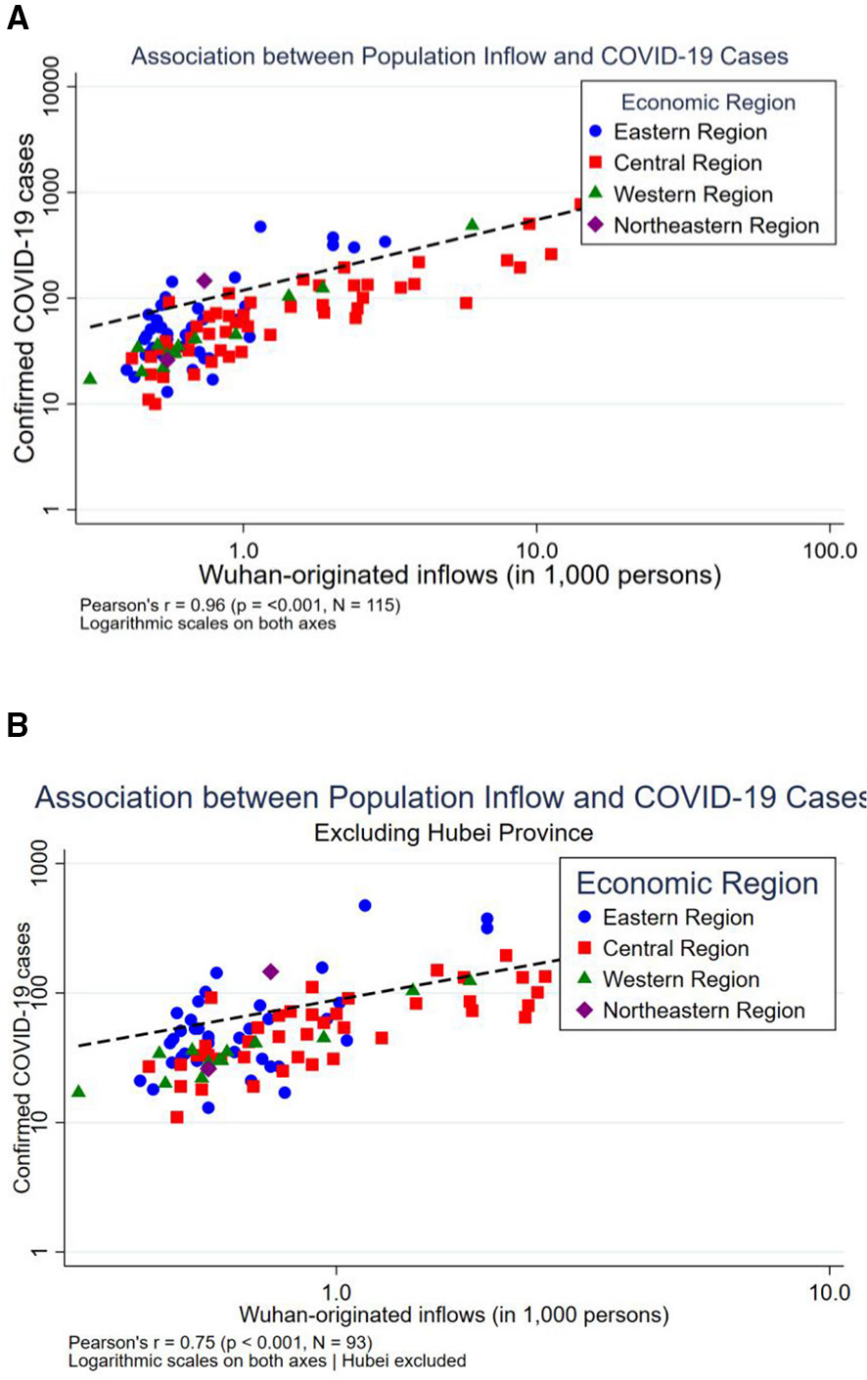
**(A)** Correlation between population movement in Wuhan Spring Festival travel rush and COVID-19 outbreak in Wuhan (full sample of cities). **(B)** Correlation between population movement in Wuhan Spring Festival travel rush and COVID-19 outbreak (deleting the sample of Hubei Province).

The results, visualized in Figure 6A, show a strong positive correlation between pre-lockdown outflows from Wuhan and the spatial distribution of confirmed cases (Pearson's *r* = 0.96, *p* < 0.001, *N* = 115). To assess whether this association is driven by cities in Hubei province, the epidemic epicenter, Figure 6B excludes the Hubei sample. The correlation remains positive and significant (*r* = 0.75, *p* < 0.001, *N* = 93), confirming that the relationship is not an artifact of Hubei's unique status. In both figures, cities are distinguished by China's four economic regions (Eastern, Central, Western, Northeastern) using distinct colors and symbols. This regional classification reveals that the positive association holds across regions rather than being concentrated in any single area. Together, these findings indicate that population inflows from Wuhan prior to January 23 were a major driver of early outbreaks in other HSR-connected cities.

Second, we employ a regression approach to estimate the effect of high-speed rail connectivity on the volume of outbound travelers from Wuhan during the Spring Festival period. The results in columns (1) and (2) of Table 13 show that cities with operational HSR services received significantly larger inflows from Wuhan, both at the provincial and city levels.

**Table 13:** Scientific basis for Wuhan lockdown: estimates based on the outflow of people from Wuhan during the Spring Festival travel rush and HSR network.

Variable	(1)	(2)	(3)	(4)
	Lnprovince_p	Lncity_p	NCP_case	NCP_case
HSR	0.413^***^ (2.83)	0.266^***^ (5.31)	3.505 (1.14)	−3.447 (−0.86)
Airport	−0.423^***^ (−3.14)	−0.0221 (−0.30)	8.778^***^ (2.92)	0.780 (0.25)
Highway intensity	1.336^***^ (12.81)	0.256^***^ (6.02)	−18.26^***^ (−3.15)	−4.224 (−1.58)
Railway	0.141 (0.47)	−0.172 (−1.57)	−13.59 (−1.35)	−0.0959 (−0.01)
Lngdp_p	−0.128 (−1.14)	0.0244 (0.41)	5.261 (1.37)	0.961 (0.33)
Lnprovince_p			22.06^***^ (4.28)	
Lncity_p				60.33^***^ (26.19)
Cons	2.792^**^ (2.38)	0.188 (0.29)	−67.91^*^ (−1.77)	−17.65 (−0.57)
*N*	293	293	293	293
*R*-squared	0.476	0.123	0.318	0.721

T-values are inside parentheses. ^*^p < 0.1, ^**^p < 0.05, ^***^p < 0.01.

Third, consider a counterfactual scenario in which city closures and travel restrictions were enforced immediately at the outbreak's onset, thus preventing large-scale population movements from Wuhan during the Spring Festival travel rush. In such a scenario, the HSR network would theoretically not contribute to COVID-19 transmission. Columns (3) and (4) of Table 13 introduce controls for provincial-level and city-specific inflows from Wuhan during the travel period. After accounting for these mobility flows, the coefficient on HSR drops substantially and becomes statistically insignificant. This finding aligns with the earlier results in Table 8, which shows that on January 24, shortly after the lockdown and before the peak of holiday migration, the difference in COVID-19 cases between HSR and non-HSR cities was minimal, averaging only 0.777 more cases in HSR-connected cities.

These findings indicate that the population mobility restrictions implemented in China were effective in containing the virus on a large scale, leading to the control of COVID-19 spread by mid-February. They also suggest that, should a similar outbreak occur in the future, earlier implementation of transport restrictions to limit population movement could further reduce virus transmission. Recent global evidence supports that containment policies significantly curb transmission, though their short-term economic impacts may vary, emphasizing the need for coordinated international responses (41). Our results align with the epidemiological framework that identifies three principal strategies for controlling infectious disease outbreaks: controlling the source of infection, interrupting transmission routes, and protecting susceptible populations.

## 6 Conclusions

This study systematically evaluates China's epidemic prevention and control measures within the context of its advanced transportation infrastructure and highly mobile urban population. Using real-time data on COVID-19 cases, HSR, city-level socioeconomic indicators, and Baidu migration records, we provide empirical evidence on the effectiveness of transport-based containment strategies, with particular focus on the Wuhan lockdown. Our principal findings are as follows.

First, the Wuhan lockdown and nationwide suspension of HSR services effectively reduced intercity transmission. Population outflows from Wuhan before the lockdown strongly predicted subsequent case counts in other HSR-connected cities, confirming that mobility from the outbreak epicenter was a key driver of early regional outbreaks. HSR connectivity significantly increased the volume of travelers departing Wuhan during the Spring Festival period, establishing it as a major channel for intercity virus spread in the initial phase.

Second, our empirical analysis employs high-frequency, multisource data and a battery of robustness checks, including alternative outcome measures, sample restrictions such as excluding Hubei Province and major administrative centers, cross-sectional analyses at different dates, placebo tests, and permutation simulations. The results remain consistent throughout, enhancing the credibility of our conclusions and helping to address the relative scarcity of causal empirical studies on epidemic control in domestic social science research.

Third, from the perspective of transportation control and city lockdowns, this research provides empirical support grounded in economics for the scientific rationale underlying China's containment strategy. Evidence indicates that measures like the Wuhan lockdown and suspension of intercity HSR services were effective in limiting the spatial and temporal diffusion of COVID-19. These findings align with and extend epidemiological and public health research on the relationship between human mobility and epidemic spread. They also offer a fact-based response to politicized narratives and unfounded criticism directed at China's early response efforts. Although early international commentary raised questions about China's measures (42), the empirical evidence presented in this study demonstrates that the Wuhan lockdown and associated transportation controls were effective in curbing the spread of the epidemic.

In broader terms, rapid urbanization and expanding transport infrastructure present a global challenge for responding to infectious disease outbreaks in a timely, evidence-based manner. Insights from this study can inform the design of mobility restriction policies in other countries facing similar public health threats. Beyond the immediate pandemic context, COVID-19 has triggered significant disruptions in global governance, trade, and geopolitical relations. Amid rising unilateralism and protectionism, the international supply chains integral to China's economy face potential reconfiguration. In this environment, integrating epidemic preparedness with long-term economic planning, including the development of a dual circulation model, will be crucial for sustaining balanced growth and enhancing resilience in an era of heightened uncertainty.

## Data Availability

The data analyzed in this study is subject to the following licenses/restrictions: the COVID-19 case data are publicly available from the websites of the National Health Commission and provincial/municipal health commissions of China. The HSR network information was compiled from public news reports and official announcements. The urban socioeconomic data are available from the China City Statistical Yearbooks. The Baidu Migration data that support the findings of this study are available from Baidu Qianxi, but restrictions apply to the availability of these raw data, which were used under license for the current study, and so are not publicly archived. Requests to access these datasets should be directed to zhangmengting@nbu.edu.cn.

## Footnotes

1 The “Eight Vertical and Eight Horizontal” is the country's medium and long-term HSR network plan. It aims to establish a 1–4 hour travel circle between adjacent large and medium-sized cities, and a 0.5–2 h travel circle within urban agglomerations. Specifically, the “Eight Vertical” corridors include the Coastal Corridor, Beijing-Shanghai Corridor, Beijing-Hong Kong (Taiwan) Corridor, Beijing-Harbin~Beijing-Hong Kong-Macao Corridor, Hohhot-Nantong Corridor, Beijing-Kunming Corridor, Baotou (Yinchuan)-Haikou Corridor, and Lanzhou (Xi'an)-Guangzhou Corridor. The “Eight Horizontal” corridors consist of the Suifenhe-Manzhouli Corridor, Beijing-Lanzhou Corridor, Qingdao-Yinchuan Corridor, Eurasian Land Bridge Corridor, Yangtze River Riverside Corridor, Shanghai-Kunming Corridor, Xiamen-Chongqing Corridor, and Guangzhou-Kunming Corridor.

2 During the Spring Festival periods in 2017 and 2018, Wuhan experienced substantial outbound population flows. In the 15 days preceding the Lunar New Year, an average of 5.2 million people departed from the city. In the 25 days following the holiday, the average daily outbound flow reached approximately 6.7 million people.

3 On February 13, according to the Hubei Provincial Health Commission, with the deepening of the understanding of COVID-19 and the accumulation of experience in diagnosis and treatment, we have added “clinical diagnosis” to the diagnostic classification of cases in Hubei Province in view of the characteristics of the epidemic in Hubei Province, which is embodied in the *Diagnostic and Treatment Program for Pneumonia Infected by New Coronavirus (Trial Implementation of the Fifth Edition)*.

4 For details, see http://qianxi.baidu.com/index.html#/.

5 In this study, a “resident city” is defined as a city where an individual has stayed for more than two months. Since each outbound migration from one city corresponds to an inbound migration to another, the national average trend of inbound migration intensity is identical to that of outbound migration intensity.

6 The World Bank's *High-Speed Railway Development in China* shows that since the opening of China's first high-speed railroad in 2008, total passenger volume has grown at an annual rate of 8.5%, dramatically changing the way people travel. Among them, the growth rate of ordinary railroads is 0.5% per year, compared with high-speed railroads, which can grow up to 81% per year. High-speed railways have a high departure density, with busy lines running 70 to 130 pairs of trains per day. The main high-speed rail lines, such as the Beijing-Shanghai and Beijing-Guangzhou lines, as well as the Yangtze River Delta and Pearl River Delta regions, have a passenger density of over 50 million per year.

7 R0 is an average that indicates the average number of peoplethat are infected by a single infected person, provided that no other factors interfere and all people are not immune. Early estimates of R0 are also dynamic, which means may change significantly as the disease progresses.

8 Zhao et al. (39) collected the average daily passenger traffic volume of various modes of transportation including buses, trains and airplanes from January 2016 to January 2019, and calculated the average daily passenger traffic volume from Wuhan to six main cities from December 16, 2019 to January 15, 2020. We then investigated the correlation between the number of passengers traveling out of Wuhan and the number of confirmed cases of COVID-19 in each location, and showed that the number of confirmed cases was significantly and positively correlated with rail transportation. Du et al. (40) estimated the number of people who flowed from Wuhan to 370 cities across China in the three weeks prior to the closure of the city based on information on ground transportation, rail transportation, and air transportation. Based on R0 = 2.56, it was shown that if 131 cities (including two cities with populations of more than two million) could be closed earlier, then there would be no confirmed cases on January 27th.
